# Antioral Cancer Effects by the Nitrated [6,6,6]Tricycles Compound (SK1) In Vitro

**DOI:** 10.3390/antiox11102072

**Published:** 2022-10-20

**Authors:** Yan-Ning Chen, Chieh-Kai Chan, Ching-Yu Yen, Jun-Ping Shiau, Meng-Yang Chang, Cheng-Chung Wang, Jiiang-Huei Jeng, Jen-Yang Tang, Hsueh-Wei Chang

**Affiliations:** 1 Graduate Institute of Medicine, College of Medicine, Kaohsiung Medical University, Kaohsiung 80708, Taiwan; 2 Institute of Chemistry, Academia Sinica, Taipei 115024, Taiwan; 3 School of Dentistry, Taipei Medical University, Taipei 11031, Taiwan; 4 Department of Oral and Maxillofacial Surgery, Chi-Mei Medical Center, Tainan 71004, Taiwan; 5 Division of Breast Oncology and Surgery, Department of Surgery, Kaohsiung Medical University Hospital, Kaohsiung Medical University, Kaohsiung 80708, Taiwan; 6 Department of Medicinal and Applied Chemistry, Kaohsiung Medical University, Kaohsiung 80708, Taiwan; 7 School of Dentistry, College of Dental Medicine, Kaohsiung Medical University, Kaohsiung 80708, Taiwan; 8 Department of Dentistry, Kaohsiung Medical University Hospital, Kaohsiung 80708, Taiwan; 9 Department of Dentistry, National Taiwan University Hospital, Taipei 100225, Taiwan; 10 Department of Radiation Oncology, Kaohsiung Medical University Hospital, Kaohsiung 80708, Taiwan; 11 School of Post-Baccalaureate Medicine, College of Medicine, Kaohsiung Medical University, Kaohsiung 80708, Taiwan; 12 Department of Biomedical Science and Environmental Biology, PhD Program in Life Science, College of Life Science, Kaohsiung Medical University, Kaohsiung 80708, Taiwan; 13 Center for Cancer Research, Kaohsiung Medical University, Kaohsiung 80708, Taiwan

**Keywords:** nitrated [6,6,6]tricycles, oral cancer cells, oxidative stress, apoptosis, DNA damage

## Abstract

A novel nitrated [6,6,6]tricycles-derived compound containing nitro, methoxy, and ispropyloxy groups, namely SK1, was developed in our previous report. However, the anticancer effects of SK1 were not assessed. Moreover, SK1 contains two nitro groups (NO_2_) and one nitrogen-oxygen (N-O) bond exhibiting the potential for oxidative stress generation, but this was not examined. The present study aimed to evaluate the antiproliferation effects and oxidative stress and its associated responses between oral cancer and normal cells. Based on the MTS assay, SK1 demonstrated more antiproliferation ability in oral cancer cells than normal cells, reversed by *N*-acetylcysteine. This suggests that SK1 causes antiproliferation effects preferentially in an oxidative stress-dependent manner. The oxidative stress-associated responses were further validated, showing higher ROS/MitoSOX burst, MMP, and GSH depletion in oral cancer cells than in normal cells. Meanwhile, SK1 caused oxidative stress-causing apoptosis, such as caspases 3/8/9, and DNA damages, such as γH2AX and 8-OHdG, to a greater extent in oral cancer cells than in normal cells. Siilar to cell viability, these oxidative stress responses were partially diminished by NAC, indicating that SK1 promoted oxidative stress-dependent responses. In conclusion, SK1 exerts oxidative stress, apoptosis, and DNA damage to a greater extent to oral cancer cells than in normal cells.

## 1. Introduction

Oral cancer is one of the leading causes of cancer death, with high morbidity in South Central Asia, Melanesia, and Taiwan [1]. Several risk factors for oral cancer include alcohol drinking, betel nut chewing, and cigarette [2,3], globally contributing to 90% of oral cancer patients [4]. Most oral cancer belongs to the oral squamous cell carcinomas occupy (>90%) [5]. The potential problems that reduce the effectiveness of chemo- and radiotherapy for oral cancer are the adverse effects [6], which may be derived from the cytotoxicity of normal tissues. Identifying anticancer drugs with low toxic effects is necessary to improve the therapeutic action.

Some natural products exhibit the benzofused bicyclo[3.3.1] skeleton, such as peniciketals A−C [7], integrastatins A and B [8], and epicoccolide A [9]. These structures were provided (Supplementary Figure S1). Integrastatins A and B and epicoccolide A exhibit anti-HIV [8] and antifungal [10] activities, but the anticancer function was unclear. Using this skeleton, we developed a novel nitrated [6,6,6]tricycles-derived compound containing nitro, methoxy, and ispropyloxy groups, namely SK1 [11]. Its IUPAC name is listed as follows: 6-Isopropoxy-5-methoxy-3,10-dinitro-12,13-dioxa-11-azatricyclo[7.3.1.0^2,7^]trideca-2,4,6,10-tetraene.

SK1 contains two nitro groups (NO_2_) and one nitrogen-oxygen (N-O) bond [11]. The NO_2_ group can function as a potential radical initiator or promoter [12]. Like O-O bond, N-O bond cleavage [13] can generate a radical structure. Furthermore, the NO_2_ group can be converted into nitrite ion (NO_2_^−^). Nitrite ion exhibits high reactivity to oxygen, generating nitrogen dioxide radical (NO_2_^•^). Moreover, NO_2_^•^ is active in reacting with biological molecules [14]. Accordingly, these NO_2_ substituents and N-O bond structures are potential mediators to generate oxidative and nitrative stress in cells [15,16].

Since oxidative stress can trigger apoptosis for antiproliferation functions [17,18], the antioral cancer effects of SK1 warrant a detailed evaluation. Moreover, the applications of several anticancer drugs are limited by their adverse effects [19]. The drug safety problem was also a concern of the present study. By selecting oral cancer and non-malignant oral cell lines, the preferential killing potential and mechanism of oral cancer cells without cytotoxic effects by SK1 were examined.

## 2. Materials and Methods

### 2.1. SK1 Preparation

The synthetic procedure of SK1 (MW = 353.08591), colorless solid; mp = 142–143 °C (recrystallized from hexanes and EtOAc), was previously described previously [11]: nitric acid (97% HNO_3_, 0.5 mL) was added stepwise to a stirred solution of 2-allyl-3-isopropoxy-4-methoxybenzaldehyde (234 mg, 1.0 mmol) in sulfuric acid (98% H_2_SO_4_, 2 mL) at room temperature for 5 min. Then, the reaction temperature was elevated to 80 °C. The heating mantle was used to provide a stable heat source. The reaction mixture was stirred at 80 °C for 24 h. The reaction was monitored by thin-layer chromatography (TLC). Next, water (10 mL) was added to the reaction mixture at room temperature, and the resulting reaction mixture was extracted with dichloromethane (3 × 20 mL). Furthermore, the combined dichloromethane layers were washed with brine (2 × 10 mL), dried with MgSO_4_, filtered with the suction funnel, and concentrated to afford the corresponding crude products by a rotavapor under reduced pressure. The crude products were purified on silica gel (hexanes/ethyl acetate = 4/1~1/1) to produce SK1 (275 mg) with a 78% yield. The ^1^H and ^13^C NMR spectra demonstrated the purity of SK1 is high (Supplementary Figure S2). As SK1 has no hydrophilic functional group, it was expected that the aqueous solubility of SK1 would be low. Consequently, SK1 was dissolved in DMSO (<0.1%) for the performance of all experiments.

### 2.2. Cell Culture and Reagents

Oral cancer (CAL 27) cell lines were derived from ATCC (Manassas, VA, USA). Oral cancer (Ca9-22 and HSC-3) cell lines were derived from JCRB Cell Bank (Osaka, Japan). The non-malignant oral cell lines, such as gingival epithelial-derived Smulow–Glickman (S–G) [20,21], were used to evaluate the drug safety of SK1. The culture medium for CAL 27, Ca9-22, HSC-3, and S–G cells was a 3:2 mixture of Dulbecco’s Modified Eagle Medium (DMEM) and F12 (Gibco, Grand Island, NY, USA), as previously mentioned [22].

Cells were treated with SK1 for 24 h. Subsequently, the MTS cell viability reagent (Promega, Madison, WI, USA) was reacted with the cell medium for 1 h to determine cell viability [23]. To address the function of oxidative stress, *N*-acetylcysteine (NAC) [24,25,26] (Sigma-Aldrich, St. Louis, MO, USA) was pretreated (10 mM, 1 h) and SK1 was posttreated for 24 h in different experiments.

### 2.3. Cell Cycle Assay

Cells were stained with 7-aminoactinmycin D (7AAD; 1 μg/mL; 30 min) (Biotium; Hayward, CA, USA) for routine flow cytometry [27] (Accuri C6, Becton-Dickinson, Mansfield, MA, USA). The subG1 population was individually counted. The summation of cell phases for G1, S, and G2/M populations was adjusted to 100%.

### 2.4. Annexin V/7AAD and Caspase 3/7 for Apoptosis Assays

According to the user instruction, cells were stained with an annexin V/7AAD kit [28] (Strong Biotech; Taipei, Taiwan) for flow-cytometry-based apoptosis detection. Moreover, apoptosis is generally detected by caspase 3/7 activity [29], which was assessed by the caspase-Glo^®^ 3/7 assay (Promega; Madison, WI, USA) for luminescence detection. Finally, the Accuri C6 flow cytometer detected annexin V/7AAD fluorescent intensity. A Luminometer (Berthold Technologies GmbH & Co., Bad Wildbad, Germany) was used to detect caspases 3/7 signal intensity. Moreover, the caspases 3/7 activity was calculated by adjusting the respective cell viability as described [29].

### 2.5. Caspases 3/8/9 for Apoptosis Assays

Flow cytometry was also applied to detect the extrinsic, intrinsic, and executor of apoptosis signaling, such as caspases 3/8/9, which were detected by peptide-specific flow cytometry [30]. In brief, PhiPhiLux-G1D2, CaspaLux8-L1D2, and CaspaLux9-M1D2 (OncoImmunin, Gaithersburg, MD, USA) were designed to react especially with activated caspases 3/8/9. Only the activated caspases 3/8/9 can digest these peptides to generate fluorescence for flow cytometry [30], according to the user’s manual.

### 2.6. ROS, Mitochondrial Superoxide (MitoSOX), and Mitochondrial Membrane Potential (MMP) for Oxidative Stress Assays

ROS, MitoSOX, and MMP levels for drug-treated cells were stained by 2′,7′-dichlorodihydrofluorescein diacetate (DCFH-DA) (Sigma-Aldrich) [23] (10 μM, 30 min), MitoSOX™ Red [23] (50 nM, 30 min), and DiOC_2_(3) [29] (Invitrogen; San Diego, CA, USA) (5 nM, 30 min). These probing for oxidative stress can generate fluorescence for flow cytometry. 

### 2.7. Cellular Antioxidant Glutathione (GSH) Assay

GSH was reacted with 5-chloromethylfluorescein diacetate (CMF-DA) (5 μM, 20 min) (Thermo Fisher Scientific, Carlsbad, CA, USA) to generate fluorescence for flow cytometry, as described previously [23].

### 2.8. γH2AX and 8-Hydroxy-2-Deoxyguanosine (8-OHdG) for DNA Damage Assays

γH2AX [31] and 8-OHdG [23], two DNA damage adducts for DNA double-strand breaks and oxidative DNA damage, were detected by flow cytometry using antibody strategy. Primary antibodies (4 °C, 1 h) for γH2AX and 8-OHdG-FITC (Santa Cruz Biotechnology) were mixed with fixed cells. Additionally, 7AAD (5 μg/mL, 30 min) was combined with a secondary antibody to γH2AX/7AAD assay. Finally, the Accuri C6 flow cytometer was used to detect γH2AX/7AAD and 8-OHdG-FITC fluorescent intensity.

### 2.9. Statistical Analysis

Data were analyzed by one-way analysis of variance (ANOVA) with HSD post hoc test by JMP 12 software (SAS Institute Inc., Cary, NC, USA). This provides low-case letters to different treatments to determine the significance in multiple comparisons. Data showing non-overlapping letters for different experiments differ significantly.

## 3. Results

### 3.1. Cell Viability of SK1 (Oral Cancer vs. Non-Malignant Cells)

**Figure 1:**
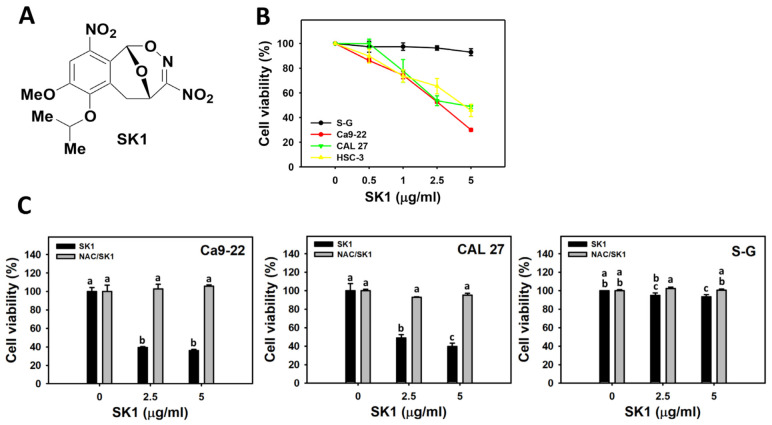
Structure and cell viability effects of SK1. (**A**) Structures of SK1. (**B**) 24 h MTS assay of SK1. Non-malignant (S–G) and oral cancer (Ca9-22, CAL 27, and HSC-3) cells were treated with SK1 for 24 h. (**C**) 24 h MTS assay of NAC/SK1. NAC/SK1 indicated that cells were pretreated with NAC and posttreated with SK1 (0 (0.1% DMSO in medium), 2.5, and 5 μg/mL for 24 h). Data, mean ± SD (*n* = 3). Statistical software provided non-overlapping low-case letters indicate significant results for multi-comparison (*p* < 0.05). In the example of Ca9-22 cells (Figure 1C), the SK1 0 μg/mL and NAC/SK1 0, showing “a” indicate nonsignificant results compared with each other because the letter was overlapping with “a”. In contrast, the SK1 2.5 μg/mL and NAC/SK1 2.5, showing “c, a, and b” indicate significant results compared with each other.

The structure of SK1 was shown (Figure 1A). The cell viability (%) of oral cancer cells (Ca9-22, CAL 27, and HSC-3) were dose-responsively inhibited by SK1 (Figure 1B), while the cell viability of non-malignant cells (S–G) was slightly changed by SK1. The IC_50_ value of SK1 at 24 h MTS assay for oral cancer cells (Ca9-22, CAL 27, and HSC-3) was 2.8 ± 0.1, 4.4 ± 1.4, and 4.4 ± 0.7 μg/mL (7.93 ± 0.35, 12.46 ± 3.89, and 12.46 ± 1.92 μM). To reduce the number of cell lines for testing, Ca9-22, CAL 27, and S–G cells were used to explore the antiproliferation mechanism in the following experiments.

To assess the impacts of oxidative stress in SK1-caused antiproliferation on oral cancer cells, pretreatments of its inhibitor (NAC) were performed. Most SK1-caused decrement of cell viability of oral cancer cells (Ca9-22 and CAL 27) were recovered close to control by NAC (NAC/SK1) (Figure 1C). 

### 3.2. Cell Cycle of SK1 (Oral Cancer vs. Non-Malignant Cells)

**Figure 2:**
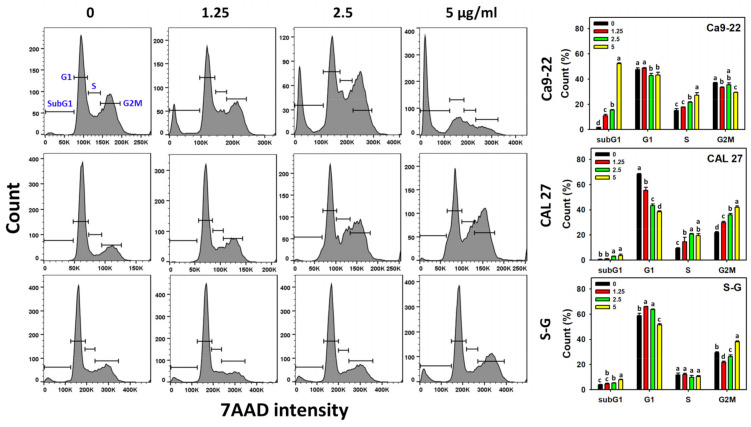
Cell cycle effects of SK1. Non-malignant (S–G) and oral cancer (Ca9-22 and CAL 27) cells were treated with SK1 (0 (0.1% DMSO in medium), 1.25, 2.5, and 5 μg/mL) for 24 h. 0, 1.25, 2.5, and 5 SK1 indicate SK1 at 0 (0.1% DMSO in medium), 1.25, 2.5, and 5 μg/mL, respectively. Data, means ± SD (*n* = 3). Statistical software provided non-overlapping low-case letters indicate significant results for multi-comparison (*p* < 0.05).

The cell cycle response to SK1 for oral cancer (Ca9-22 and CAL 27) and non-malignant (S–G) cells was examined by flow cytometry. The population of subG1 dramatically increased after SK1 treatment in Ca9-22 cells but few in CAL 27 and S–G cells (Figure 2). Generally, SK1 induced more G1 decrements and G2/M increments in oral cancer cells than in S–G cells.

### 3.3. Apoptosis (Annexin V) of SK1 (Oral Cancer vs. Non-Malignant Cells)

**Figure 3:**
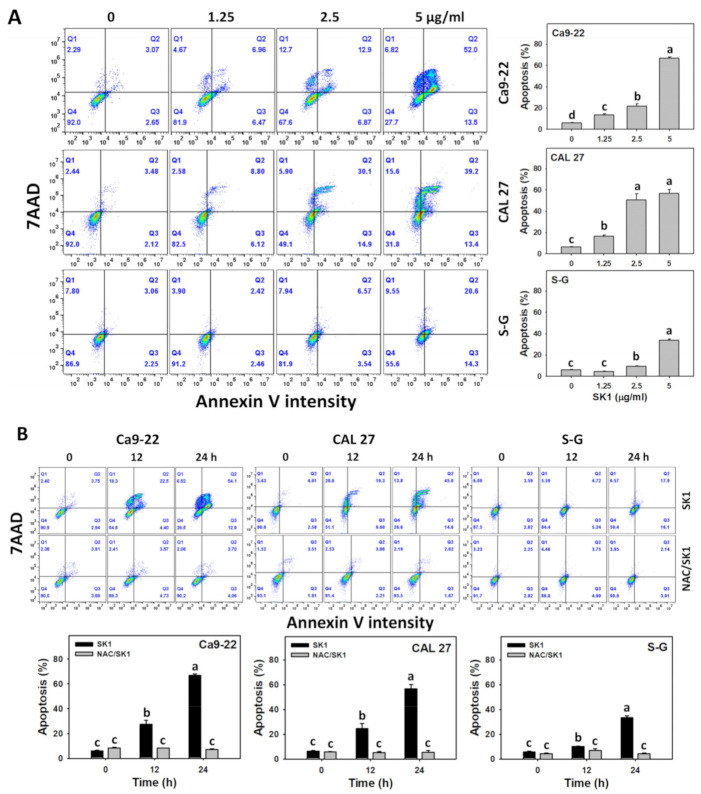
Apoptosis effects (annexin V) of SK1. (**A**) Annexin V/7AAD assays of SK1. Non-malignant (S–G) and oral cancer (Ca9-22 and CAL 27) cells were treated with SK1 (0 (0.1% DMSO in medium), 1.25, 2.5, and 5 μg/mL) for 24 h. (**B**) Annexin V/7AAD assays of NAC/SK1. NAC/SK1 indicated that cells were pretreated with NAC and posttreated with SK1 5 μg/mL for 0, 12, and 24 h. Apoptosis (%) is counted for percentage of annexin V (+)/7AAD (+/−)(%). Data, mean ± SD (*n* = 3). Statistical software provided non-overlapping low-case letters indicate significant results for multi-comparison (*p* < 0.05).

The apoptosis effects were assessed by annexin V/7AAD assays. For dose effects, SK1 caused more annexin V increment in Ca9-22 and CAL 27 cells than S–G cells (Figure 3A). As mentioned above, NAC, a ROS inhibitor, also affected the SK1-caused antiproliferation; the contribution of oxidative stress acting on apoptosis was assessed by annexin V/7AAD assays. For time effects, SK1 caused more annexin V increment in oral cancer cells than S–G cells (Figure 3B). These SK1-caused annexin V increments were suppressed by NAC (NAC/SK1 treatment).

### 3.4. Apoptosis (Caspases 3 and 3/7) of SK1 (Oral Cancer vs. Non-Malignant Cells)

**Figure 4:**
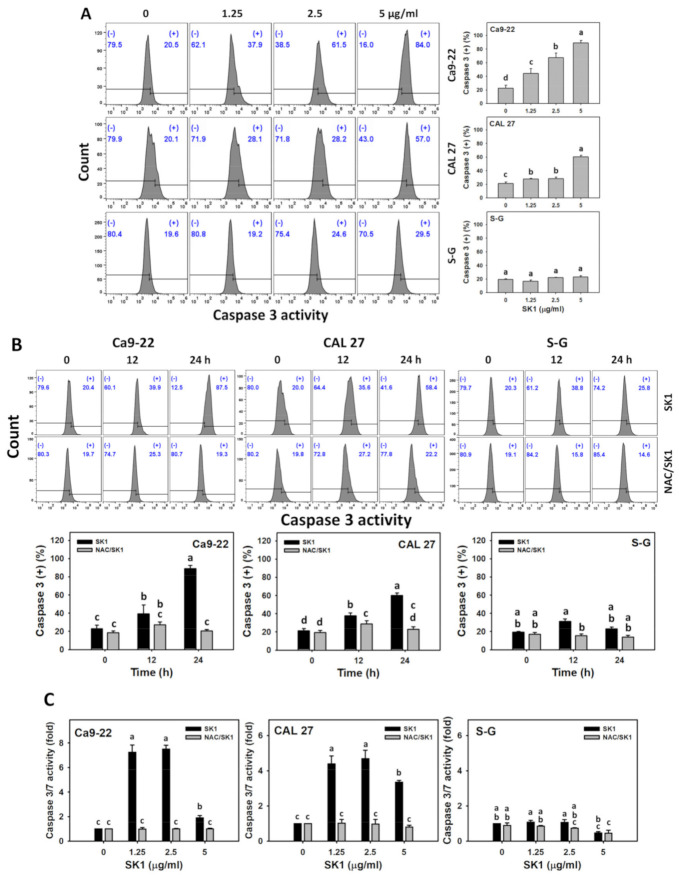
Apoptosis effects (flow cytometry-detected caspase 3 and luminescence-detected caspase 3/7 assays) of SK1. (**A**) Caspase 3 assay of SK1. Non-malignant (S–G) and oral cancer (Ca9-22 and CAL 27) cells were treated with SK1 (0 (0.1% DMSO in medium), 1.25, 2.5, and 5 μg/mL) for 24 h. (**B**) Caspase 3 assay of NAC/SK1. NAC/SK1 indicated that cells were pretreated with NAC and posttreated with SK1 5 μg/mL for 0, 12, and 24 h. (**C**) Caspase 3/7 assay of NAC/SK1. NAC/SK1 indicated that cells were pretreated with NAC and posttreated with SK1 1.25, 2.5, and 5 μg/mL for 24 h. (+) inserted at the histogram is counted for the percentage of caspase 3 (+)(%). Data, mean ± SD (*n* = 3). Statistical software provided non-overlapping low-case letters indicate significant results for multi-comparison (*p* < 0.05).

The apoptosis signaling for caspase activation was analyzed by flow cytometry-detected caspases 3 and luminescence-detected caspase 3/7 assays. For dose effects, SK1 caused more caspase 3 and caspase 3/7 increments in Ca9-22 and CAL 27 cells than that of S–G cells (Figure 4A,C).

The contribution of oxidative stress acting on apoptosis signaling was assessed by caspases 3 and 3/7 assays under NAC pretreatment. For time effects, SK1 caused more caspase 3 and caspase 3/7 increments in oral cancer cells than that of S–G cells (Figure 4B,C). These SK1-caused caspase 3 increments were suppressed by NAC (NAC/SK1 treatment), particularly for 12 and 24 h for oral cancer and S–G cells. These SK1-caused caspase 3/7 increments for 1.25, 2.5, and 5 μg/mL SK1 were suppressed by NAC (NAC/SK1 treatment), particularly for 24 h for oral cancer cells.

### 3.5. Apoptosis (Caspases 8 and 9) of SK1 (Oral Cancer vs. Non-Malignant Cells)

**Figure 5:**
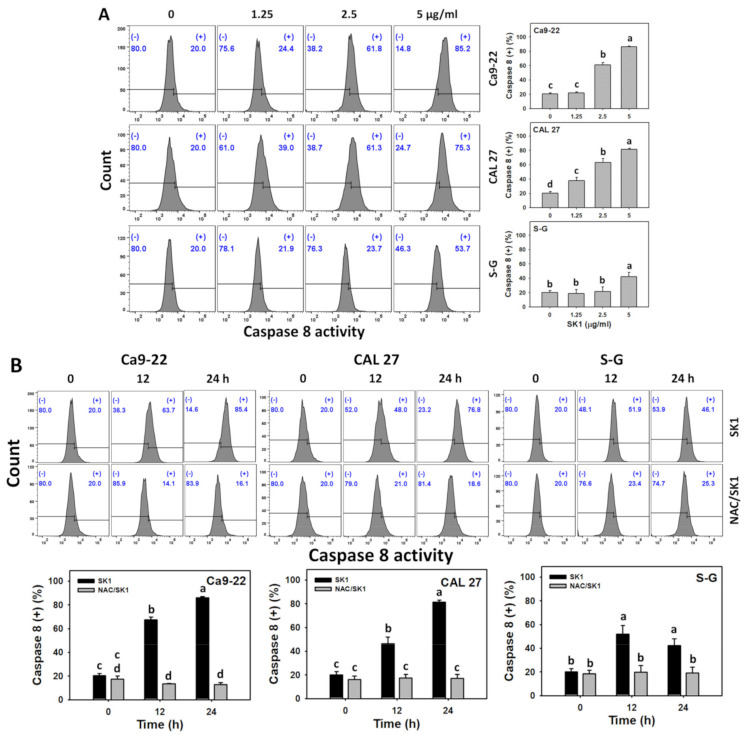

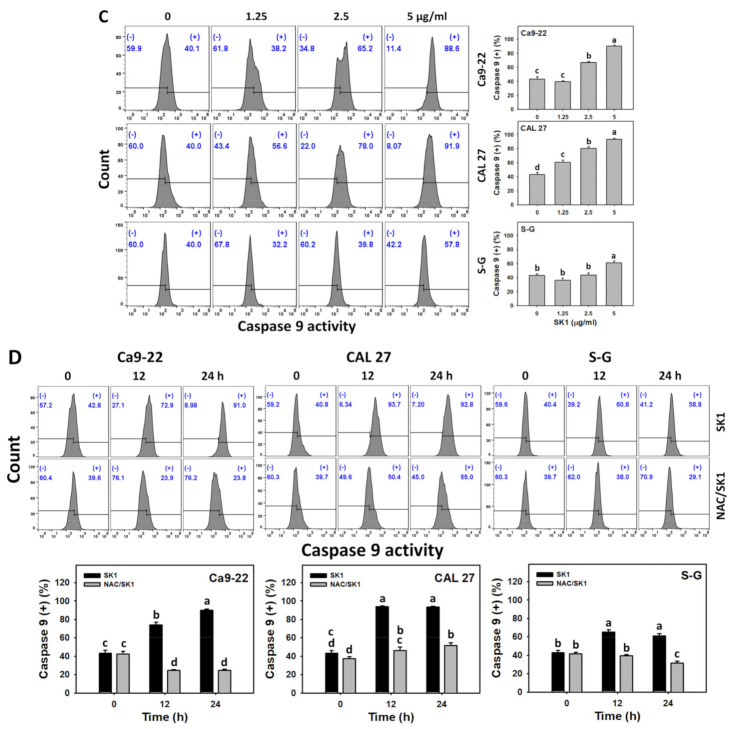
Apoptosis effects (Caspase 8 and 9) of SK1. (**A**,**C**) Caspase 8 and caspase 9 assays of SK1. Non-malignant (S–G) and oral cancer (Ca9-22 and CAL 27) cells were treated with SK1 (0 (0.1% DMSO in medium), 1.25, 2.5, and 5 μg/mL) for 24 h. (**B**,**D**) Caspase 8 and caspase 9 assays of NAC/SK1. NAC/SK1 indicated that cells were pretreated with NAC and posttreated with SK1 5 μg/mL for 0, 12, and 24 h. (+) inserted at the histogram is counted for the percentage of caspase 8 or caspase 9 (+)(%). Data, mean ± SD (*n* = 3). Statistical software provided non-overlapping low-case letters indicate significant results for multi-comparison (*p* < 0.05).

Since the executor (caspases 3 and 3/7) of apoptosis signaling was activated, as mentioned above, the impacts of the upstream extrinsic and intrinsic caspases by SK1 were assessed. For dose effects, SK1 caused more caspases 8 and 9 increments in Ca9-22 and CAL 27 cells than that of S–G cells (Figure 5A,C).

The contribution of oxidative stress acting on apoptosis signaling was assessed by caspases 8 and 9 assays under NAC pretreatment. For time effects, SK1 caused more caspases 8 and 9 increments in oral cancer cells than that of S–G cells (Figure 5B,D).

### 3.6. Oxidative Stress (ROS and MitoSOX) of SK1 (Oral Cancer vs. Non-Malignant Cells) 

**Figure 6:**
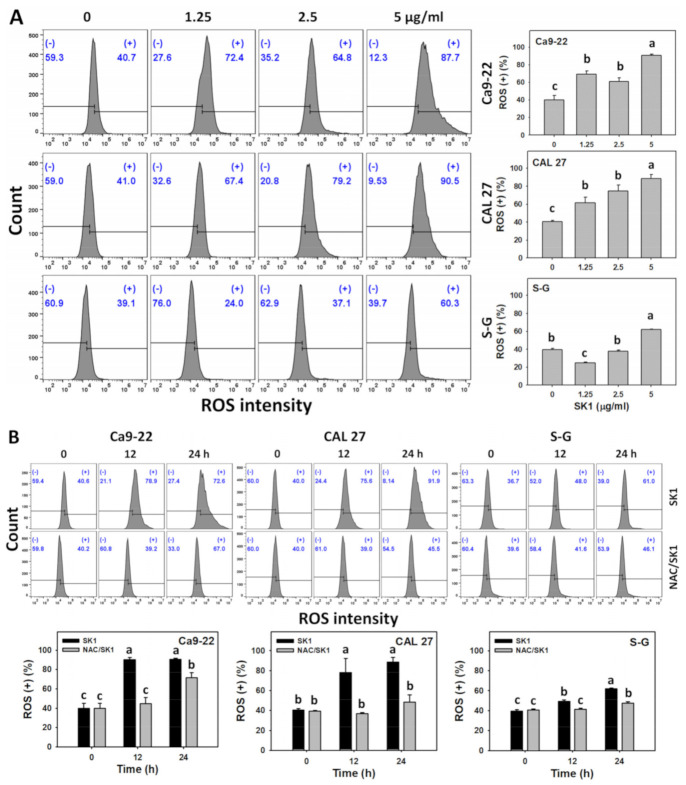

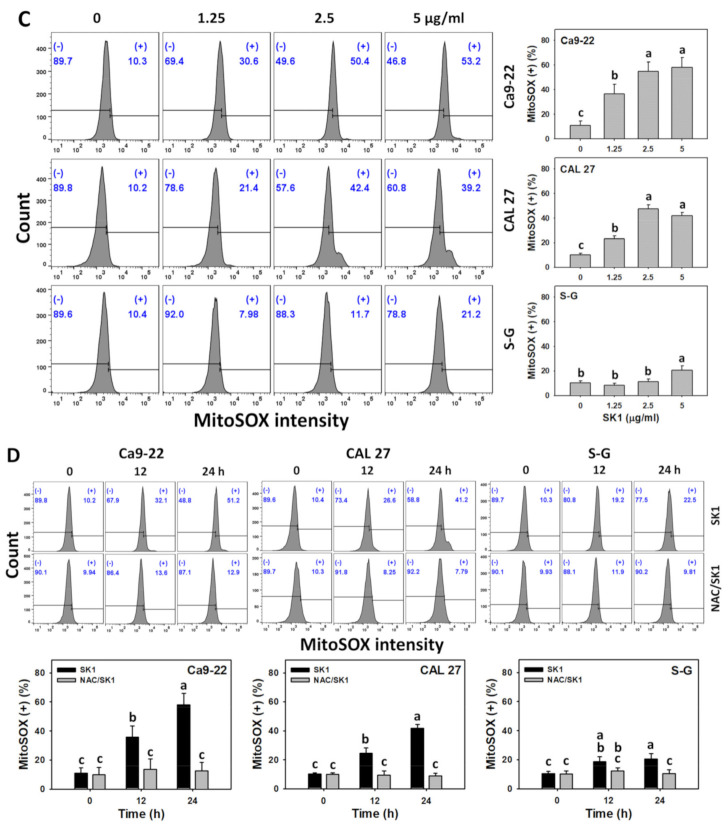
Oxidative stress effects (ROS and MitoSOX) of SK1. (**A**,**C**) ROS and MitoSOX assays of SK1. Non-malignant (S–G) and oral cancer (Ca9-22 and CAL 27) cells were treated with SK1 (0 (0.1% DMSO in medium), 1.25, 2.5, and 5 μg/mL) for 24 h. (**B**,**D**) ROS and MitoSOX assays of NAC/SK1. NAC/SK1 indicated that cells were pretreated with NAC and posttreated with SK1 5 μg/mL for 0, 12, and 24 h. (+) inserted at the histogram is counted for the percentage of ROS and MitoSOX (+)(%). Data, mean ± SD (*n* = 3). Statistical software provided non-overlapping low-case letters indicate significant results for multi-comparison (*p* < 0.05).

Since NAC effects on cell viability and apoptosis are mentioned above, the induction of oxidative stress by SK1 was assessed. For dose effects, SK1 caused more ROS and MitoSOX increments in Ca9-22 and CAL 27 cells than that of S–G cells (Figure 6A,C).

The involvement of oxidative stress was assessed by ROS and MitoSOX assays under NAC pretreatment. For time effects, SK1 caused more ROS and MitoSOX increments in oral cancer cells than that of S–G cells (Figure 6B,D).

### 3.7. Oxidative Stress (MMP) of SK1 (Oral Cancer vs. Non-Malignant Cells)

**Figure 7:**
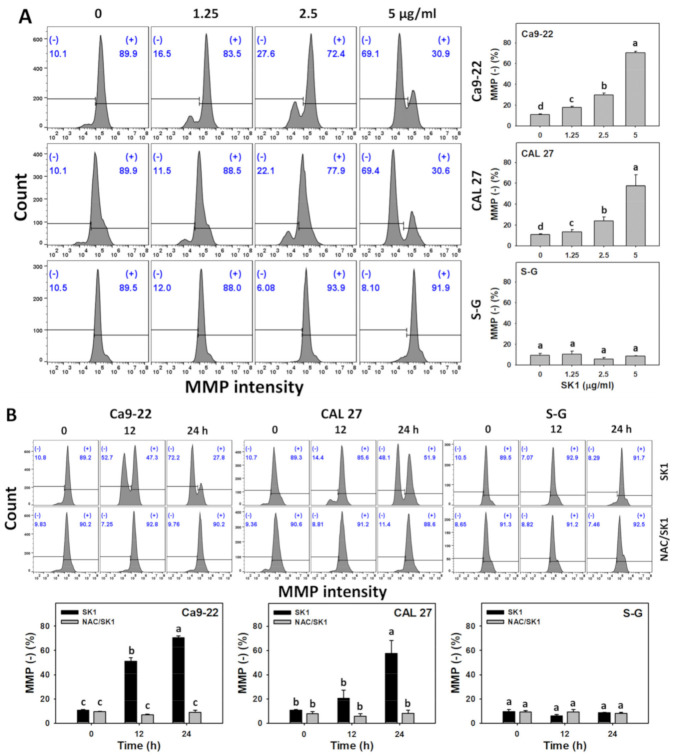
Oxidative stress effects (MMP) of SK1. (**A**) MMP assays of SK1. Non-malignant (S–G) and oral cancer (Ca9-22 and CAL 27) cells were treated with SK1 (0 (0.1% DMSO in medium), 1.25, 2.5, and 5 μg/mL) for 24 h. (**B**) MMP assays of NAC/SK1. NAC/SK1 indicated that cells were pretreated with NAC and posttreated with SK1 5 μg/mL for 0, 12, and 24 h. (−) inserted at the histogram is counted for the percentage of MMP (−)(%). Data, mean ± SD (*n* = 3). Statistical software provided non-overlapping low-case letters indicate significant results for multi-comparison (*p* < 0.05).

Since oxidative stress (ROS and MitoSOX) effects are mentioned above, the induction of other oxidative stress, such as MMP by SK1, was assessed. For dose effects, SK1 caused more MMP decrements in Ca9-22 and CAL 27 cells than in S–G cells (Figure 7A).

The involvement of oxidative stress was further assessed by MMP assays under NAC pretreatment. For time effects, SK1 caused more MMP decrements in oral cancer cells than in S–G cells (Figure 7B).

### 3.8. Cellular Antioxidant (GSH) of SK1 (Oral Cancer vs. Non-Malignant Cells)

**Figure 8:**
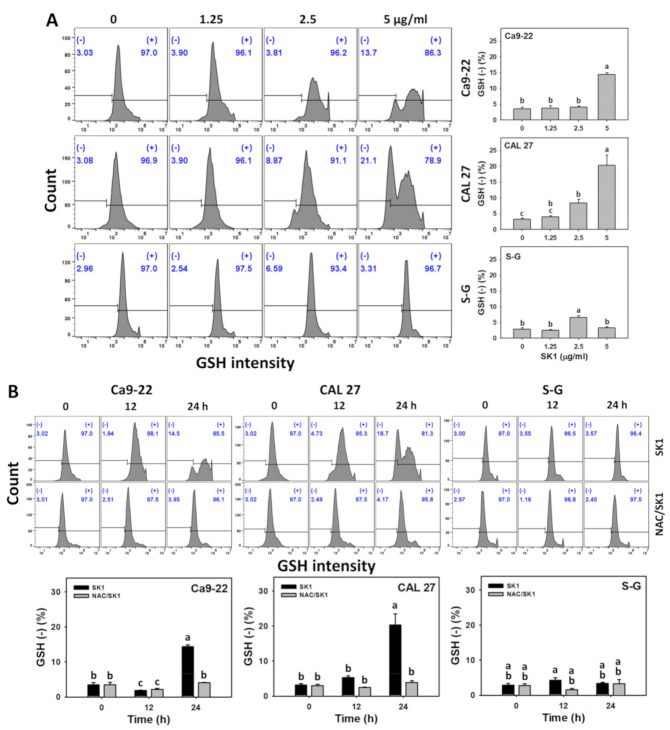
Antioxidant responses (GSH) of SK1. (**A**) GSH assays of SK1. Non-malignant (S–G) and oral cancer (Ca9-22 and CAL 27) cells were treated with SK1 (0 (0.1% DMSO in medium), 1.25, 2.5, and 5 μg/mL) for 24 h. (**B**) GSH assays of NAC/SK1. NAC/SK1 indicated that cells were pretreated with NAC and posttreated with SK1 5 μg/mL for 0, 12, and 24 h. (−) inserted at the histogram is counted for the percentage of GSH (−)(%). Data, mean ± SD (*n* = 3). Statistical software provided non-overlapping low-case letters indicate significant results for multi-comparison (*p* < 0.05).

Since oxidative stress effects are mentioned above, the antioxidant response, such as GSH change by SK1, was assessed. For dose effects, SK1 caused more GSH decrements in Ca9-22 and CAL 27 cells than in S–G cells (Figure 8A).

The involvement of cellular antioxidant response was further assessed by GSH assays under NAC pretreatment. For time effects, SK1 caused more GSH decrements in oral cancer cells than in S–G cells (Figure 8B).

### 3.9. DNA Damages (γH2AX and 8-OHdG) of SK1 (Oral Cancer vs. Non-Malignant Cells)

**Figure 9:**
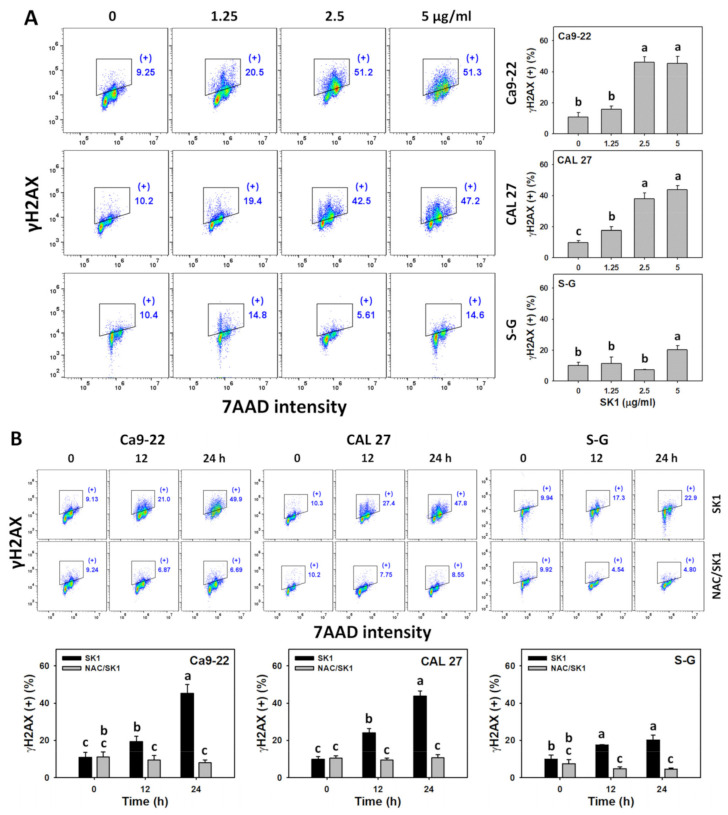
DNA damage effects (γH2AX) of SK1. (**A**) γH2AX assays of SK1. Non-malignant (S–G) and oral cancer (Ca9-22 and CAL 27) cells were treated with SK1 (0 (0.1% DMSO in medium), 1.25, 2.5, and 5 μg/mL) for 24 h. (**B**) γH2AX assays of NAC/SK1. NAC/SK1 indicated that cells were pretreated with NAC and posttreated with SK1 5 μg/mL for 0, 12, and 24 h. (+) inserted at the histogram is counted for the percentage of γH2AX (+)(%). Data, mean ± SD (*n* = 3). Statistical software provided non-overlapping low-case letters indicate significant results for multi-comparison (*p* < 0.05).

**Figure 10:**
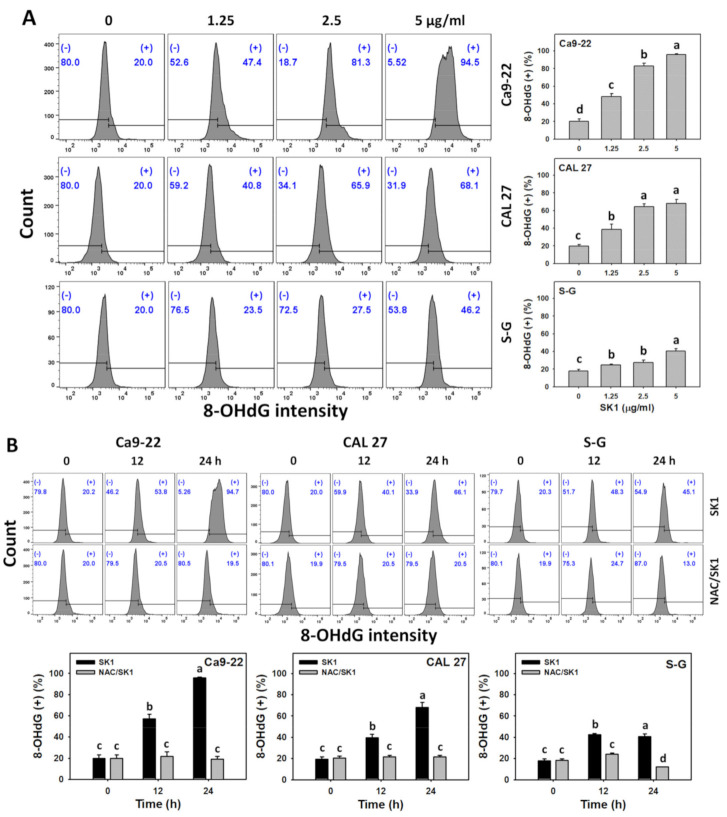
DNA damage effects (8-OHdG) of SK1. (**A**) 8-OHdG assays of SK1. Non-malignant (S–G) and oral cancer (Ca9-22 and CAL 27) cells were treated with SK1 (0 (0.1% DMSO in medium), 1.25, 2.5, and 5 μg/mL) for 24 h. (**B**) 8-OHdG assays of NAC/SK1. NAC/SK1 indicated that cells were pretreated with NAC and posttreated with SK1 5 μg/mL for 0, 12, and 24 h. (+) inserted at the histogram is counted for the percentage of 8-OHdG (+)(%). Data, mean ± SD (*n* = 3). Statistical software provided non-overlapping low-case letters indicate significant results for multi-comparison (*p* < 0.05).

Oxidative stress improves DNA damage [32]. Hence, γH2AX and 8-OHdG, two common DNA damage markers, were assessed. For dose effects, SK1 caused more γH2AX and 8-OHdG increments in Ca9-22 and CAL 27 cells than in S–G cells (Figure 9 A and Figure 10A).

The contribution of oxidative stress effects acting on DNA damage was assessed by γH2AX and 8-OHdG assays under NAC pretreatment. For time effects, SK1 caused more γH2AX and 8-OHdG increments in oral cancer cells than in S–G cells (Figure 9B and Figure 10B).

## 4. Discussion

The present study evaluated the antiproliferation impact of SK1 on oral cancer and non-malignant cells. Several antiproliferation mechanisms of SK1 were explored and discussed.

The nitro group-containing drugs exhibit antiproliferation effects against several cancer cells [33]. For example, azithromycin showed an IC_50_ value of 200 µM at 24 h SRB assay for colon cancer cells (HCT-116) [34]. Nifurtimox showed an IC_50_ value of 17.17 μg/mL at 48 h MTS assay for neuroblastoma LA-N2 cells [35]. Compounds with the bicyclo[3.3.1] skeleton also belong to the nitro-group-containing drugs and may exhibit anticancer effects. For example, peniciketal B, a spiroketal containing a benzo-fused bicyclo[3.3.1] skeleton [36], showed an IC_50_ value of 6.8 μM for A549 lung cancer cells but showed low cytotoxicity to normal lung fibroblast cells (IMR90) [37]. For comparison, the IC_50_ value of SK1 at 24 h MTS assay for oral cancer cells (Ca9-22, CAL 27, and HSC-3) was 2.8, 4.4, and 4.4 μg/mL (7.93, 12.46, and 12.46 μM) without cytotoxic effects to non-malignant oral cells. For clinical drugs, 5-fluorouracil showed the IC_50_ of 95.6 μM at 24 h Alamar blue assay for oral cancer cells (CAL 27) [38] and was associated with adverse effects [39]. Consequently, SK1 demonstrated the preferential antiproliferation impact with high drug sensitivity to oral cancer cells without harmful effects on non-malignant (S–G) cells. Notably, the apoptosis (both early and late) of S–G cells after SK1 treatment slightly increases after 24 h. Thus, SK1 impact on viability of S–G probably needs a longer incubation time. This warrants a detailed evaluation of proliferation for more prolonged treatment in the future.

The compounds with the nitro group (NO_2_) or N-O bond are potential mediators to generate oxidative and nitrative stress in cells [15,16]. SK1 containing two NO_2_ groups and one N-O bond structure exhibits the potential to generate radicals [12] such as NO_2_^•^. It warrants a detailed assessment of oxidative stress response to SK1. In the present study, the oxidative stress induction of SK1 was confirmed by ROS, MitoSOX, and MMP assays (Figure 6 and Figure 7). Moreover, this oxidative stress also contributed to the antiproliferation of SK1 because NAC could rescue this antiproliferation effect (Figure 1). Notably, SK1 generated more oxidative stress in oral cancer than non-malignant cells. Therefore, SK1 induces oxidative stress to exert antiproliferation to oral cancer cells but not non-malignant cells. However, the protective effect of NAC could be a consequence of a direct binding to SK1. To exclude the direct binding of NAC to SK1, an additional experiment of cell viability needs to be examined in the future.

The oxidative stress level is balanced by the cellular antioxidant system [40,41]. When antioxidant system was downregulated, the cellular oxidative stress was upregulated. For example, marine natural product fucoidan inhibits GSH expressions, leading to oxidative stress bursts in oral cancer cells [23]. Similarly, SK1 downregulated more GSH levels in oral cancer cells at 24 h than in non-malignant cells (Figure 8). Therefore, modulating antioxidant signaling contributed to SK1-promoting oxidative stress in oral cancer cells.

In general, cancer cells show higher ROS levels than normal cells [42,43]. When oxidative stress-generating anticancer drugs provide the exogenous ROS exceeding the threshold of cancer cells, it causes cancer cell antiproliferation; however, this ROS may be tolerant due to normal cells remaining healthy [42,43]. This ROS-modulating strategy for preferential antiproliferation against cancer cells has been applied to several anticancer drugs such as Motexafin gadolinium [44], β-lapachone [45], fucoidan [23], and manoalide [22]. This rationale may partly explain why SK1 exhibits preferential oxidative stress activation in oral cancer cells rather than non-malignant cells.

Moreover, NO_2_^•^ is active in a chemical reaction with biological molecules [14]. For example, NO_2_^•^-promote oxidative damage in the side chain and backbone of peptides. NO_2_^•^ attacks cholesterol to generate 6-nitrocholesterol. NO_2_^•^ also shows a fast reaction with the guanine radical cation [46,47,48], forming 8-nitroguanine to cause DNA damage [49]. 

With the involvement of in situ formed NO_2_^•^, oxidation and nitration reactions in DNA also promote the formation of 8-oxo-7,8-dihydroguanine [50]. 8-Oxo-7,8-dihydroguanine and its isomer 8-OHdG are the most common oxidative DNA damage product because its C-8 is highly reactive to ROS [51]. In the present study, the DNA damage effects of SK1 were validated by γH2AX, a DNA double-strand break marker (Figure 9). Moreover, the oxidative DNA damage effects of SK1 in oral cancer cells were confirmed by 8-OHdG (Figure 10).

Many anticancer drugs were developed to provoke oxidative stress to induce apoptosis [25,52,53,54,55]. Similarly, SK1 caused apoptosis and activated caspases 3, 8, and 9 to demonstrate that SK1 activates both extrinsic and intrinsic caspase signaling in oral cancer cells, triggering apoptosis.

In addition to antiproliferation, NAC blocks apoptosis, caspases 3/8/9 signaling, oxidative stress (ROS, MitoSOX, and MMP), antioxidant response, and DNA damage. This demonstrated that SK1 induced antiproliferation by modulating oxidative stress-related responses. Moreover, SK1 generated higher oxidative stress-related responses in oral cancer cells than in non-malignant cells, contributing to the finding that SK1 preferentially kills oral cancer cells compared to non-malignant cells.

## 5. Conclusions

Benzofused bicyclo[3.3.1] skeleton was used to synthesize a novel nitrated [6,6,6]tricycles-derived compound containing nitro, methoxy, and ispropyloxy groups, namely SK1 [11]. However, the anticancer effects of SK1 were not assessed. SK1 demonstrated antiproliferation to a greater extent in oral cancer cells than in normal cells, depending on oxidative stress. Consequently, SK1 promoted oxidative stress-associated responses that were higher in oral cancer cells than in normal cells, such as ROS/MitoSOX burst, MMP, and GSH depletion. Moreover, SK1 triggered more oxidative stress-causing apoptosis, such as caspases 3/8/9, and DNA damages, such as γH2AX and 8-OHdG, in oral cancer cells than normal cells. NAC partially diminished all these oxidative stress responses, demonstrating that SK1 caused these oxidative stress responses to be ROS-dependent, contributing to the antiproliferation of oral cancer cells but not normal cells. Exploring these antioral cancer mechanisms may accelerate the application of SK1 in oral cancer treatment.

## Data Availability

All data are included within the article and Supplementary Materials.

## Supplementary Materials

The following are available online at https://www.mdpi.com/article/10.3390/antiox11102072/s1, Figure S1: Structures of natural products mentioned in the Introduction section; Figure S2: ^1^H and ^13^C NMR spectra of SK1.
