# Supplementary material for: Antioral Cancer Effects by the Nitrated [6,6,6]Tricycles Compound (SK1) In Vitro
Source: Antioxidants (Basel). 2022 Oct 20;11(10):2072. doi: 10.3390/antiox11102072 (PMC9598307; doi:10.3390/antiox11102072)
Supplement: Supplementary file 1 [file antioxidants-11-02072-s001.zip › antioxidants-1953126-supplementary.pdf]

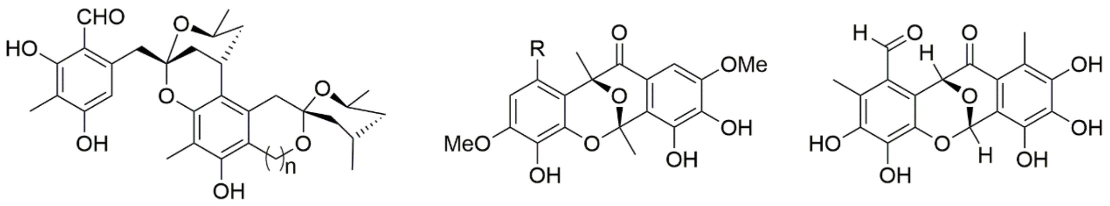

peniniketal A: n = 1, R= OH
peniniketal B: n = 1, R= H
peniniketal C: n = 0, R= OH

integrastatin A: R = CH_2OH
integrastatin B: R = CHO

epicoccolide A

Supplementary Figure S1. Structure of peniciketals A−C, integrastatins A and B, and epicoccolide A mentioned in the introduction.

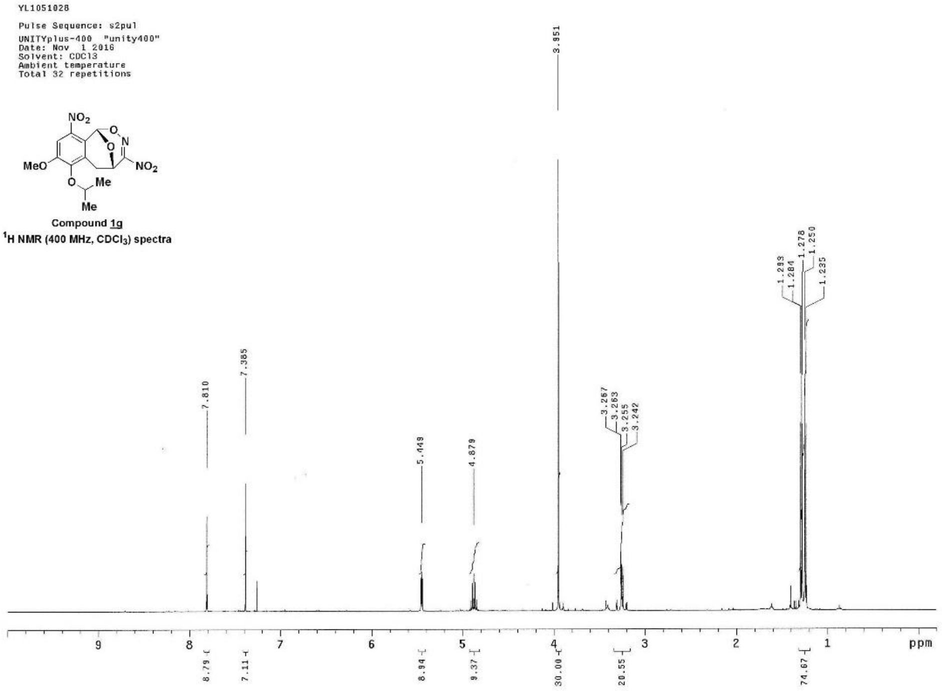

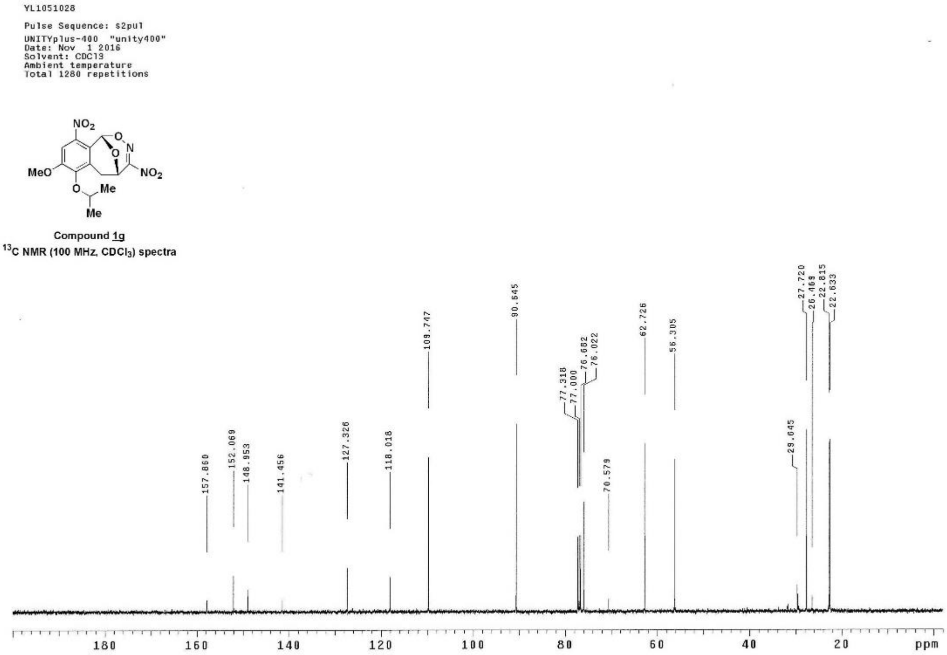

Supplementary Figure S2. ^{1}H (top) and ^{13}C (bottom) NMR spectra of SK1 (in CDCl3, 400/100 MHz).
